# Excess mortality and associated community risk factors related to hurricane Maria in Puerto Rico

**DOI:** 10.1088/2752-5309/adac03

**Published:** 2025-02-03

**Authors:** Kristen N Cowan, Diego E Zavala, Erick Suarez, José A Lopez-Rodriguez, Omar Alvarez

**Affiliations:** 1 Department of Epidemiology, University of North Carolina Gillings School of Global Public Health, Chapel Hill, NC, United States of America; 2 Public Health Program, Ponce Health Sciences University, Ponce, PR, United States of America; 3 Department of Biostatistics and Epidemiology, University of Puerto Rico Medical Sciences Campus, San Juan, PR, United States of America; 4 Demographic Consultant, San Juan, PR, United States of America; 5 Puerto Rico Planning Board, San Juan, PR, United States of America

**Keywords:** hurricane, vulnerability, mortality, spatial epidemiology, disasters

## Abstract

Background: In the 6 months following Hurricane Maria the number of people who died from the hurricane was much higher than was initially estimated from death certificates. Disruption of health care services and displacement led to the exacerbation of pre-existing chronic diseases. The objectives of this study were to (1) estimate the excess deaths in Puerto Rico in the 6 months following Maria, (2) identify geographical areas experiencing higher risk of chronic disease mortality following Maria and (3) identify community-level vulnerability characteristics associated with some communities being at higher risk of increased chronic disease mortality after Maria. Methods: Death records were obtained from Puerto Rico’s Department of Health Demographic Registry. Mortality risks per 100 000 were calculated for chronic disease categories and all-cause mortality for the 6 months following Maria and the same months in the year before. Geospatial analysis using Getis–Ord Gi* Statistic was used to determine if mortality clusters of 6 month mortality risk following hurricane Maria by census tract were statistically significant. Multinomial logistic regression was used to model the association between census tract level social vulnerability and being classified as higher or sustained risk of mortality in the 6 months following Hurricane Maria compared to the previous year’s mortality risk. Odds ratios and 95% confidence intervals were estimated to measure associations between social vulnerability and mortality risk. Results: In the 6 months following Maria there were increases in mortality risk for cardiovascular disease, Alzheimer’s, diabetes, sepsis, chronic respiratory disease, hypertension and all-cause mortality. Examining community level characteristics associated with vulnerability to disasters, neighborhoods with higher proportion of people 65 and older, higher proportion of houses being multiunit structures and higher proportion of households with no vehicle, in comparison to other neighborhoods in Puerto Rico,were more likely to have sustained high risk for mortality before and after Maria or increased risk of being a hot spot for chronic disease mortality after Maria.

## 1. Introduction

Hurricane Maria made landfall in Puerto Rico on 20th September, 2017 as a category 4 hurricane devastating the island infrastructure and resulting in up to 37.9 inches of total rainfall, and 6–9 feet of flooding and mud slides across the island [1]. The first official report of 64 deaths following hurricane Maria was shown to be a gross underestimate since the United States Centers for Disease control and prevention (CDC) certification of deaths related to disasters protocol was not implemented in Puerto Rico prior to hurricane Maria to document direct and indirect causes of deaths attributed to a disaster [2]. A study commissioned by the Puerto Rican government suggested the death toll be revised upward to 2 975 deaths attributable to hurricane Maria [3]. In the year that followed this disaster, determining the number of people who died directly or indirectly from the impact of the hurricane was the subject of debate and various methods were used to estimate mortality in the short- and long-term following Maria [3–7]. One study that reviewed all of post-Maria mortality estimates in Puerto Rico with varying statistical methods and periods of analyses concluded that considering population displacement after the disaster, estimates of excess mortality between September 2017 and early spring of 2018, between 3000–3500 excess deaths may have occurred in Puerto Rico [8].

Most of these studies done after hurricane Maria provided overall estimate of excess death counts, however it is well-documented that individuals with chronic diseases are unable to adequately control their health conditions after a disaster which can lead to critical disease exacerbations [8] and possibly greater risk of death. After hurricane Maria, many people living with chronic diseases on the island had difficulties accessing appropriate medical care, particularly those who needed dialysis. Depending on the socioeconomic status and underlying health issues of people living in the areas most affected by a storm, hurricanes can lead to large increases in morbidity and mortality associated with infectious diseases, environmental hazards, injuries, and chronic diseases [9–11]. In Puerto Rico almost half of the population is covered by Medicaid [12] and many of them are living with at least one chronic disease with 42% of the Puerto Rican population having hypertension [13]. Additionally, many patients with end-stage renal disease in Puerto Rico receive dialysis at one of two locations which care for 2000–4000 patients each [14]. The severe infrastructure damage caused by the hurricane eliminated access to the health care that many patients needed to maintain control of their chronic diseases. In the months following hurricane Maria, access to health care continued to be limited and environmental hazards such as flooding and mold growth may have also contributed to death and disease among Puerto Ricans.

Previous studies have not examined community-level characteristics associated with chronic disease mortality risk after Hurricane Maria in Puerto Rico. The objectives of this study were to [1] estimate the excess deaths for the most common causes of death in Puerto Rico in the 6 months following hurricane Maria [2], identify geographical areas experiencing higher risk of chronic disease mortality following hurricane Maria and [3] identify community level vulnerability characteristics that are associated with communities having a higher risk of observing an increase in chronic disease mortality after hurricane Maria.

## 2. Methods

### 2.1. Data sources and classifications

Death certificate records from 13 September 2017–31 March 2018 and for the same dates in the year before hurricane Maria, that is 13 September 2016–31 March 2017, were obtained from Puerto Rico’s Department of Health Demographic Registry. Death certificate data for the week prior to the hurricane was included to account for deaths due to injuries or other external causes resulting from people preparing for the impact of the hurricane. The death certificate data includes sociodemographic information such as age, sex, race, marital status, place of residence, residential area, place of death, education, occupation, type of death, and Hispanic origin among others. In addition, the database contains important information for public health officials such as the variables underlying the immediate cause of death. All causes of death were grouped using the National Center for Health Statistics classification including categories for the most common chronic health conditions in Puerto Rico [15]. Cause of death was recoded to fall into one of the following causes of death: Alzheimer’s disease, cardiovascular disease (CVD), end stage renal disease, chronic obstructive pulmonary disease, mental health conditions, sepsis, asthma, hypertension, cancer, chronic respiratory disease, suicide, and all other causes. Decedent addresses for a subset of chronic disease categories were geocoded and aggregated at the census tract level to examine counts for chronic disease mortality before and after hurricane Maria.

Data on Puerto Rico population estimates were obtained from the Census Bureau for 2016–2018 [16]. To obtain an estimate of the population in pre-Maria time period, an average of the 2016 and 2017 population was calculated and the same was done for the post-Maria time period for 2017 and 2018. Risks reported in table 2 were calculated using these population estimates as the denominators.

Data on social vulnerability by census tracts in Puerto Rico was obtained from the Centers for Disease CDC /Agency for Toxic Substances and Disease Registry 2018 Social Vulnerability Index [17]. The social vulnerability index uses 15 census tract variables and is categorized into four categories [1], Socioeconomic [2], Household Composition and Disability [3], Minority Status and Language and [4] Housing Type. Census tracts are ranked based on percentile for each social vulnerability category and overall social vulnerability. The social vulnerability index ranges from 0 to 1 with 0 being the least vulnerable and 1 being the most vulnerable. The social vulnerability indicators are described further in supplemental table 1. For this study, only three social vulnerability themes (socioeconomic, household composition/disability, and housing type) were used as the subtheme on minority status and language is not applicable to Puerto Rico, therefore results are only presented for the individual themes examined and not the overall Social Vulnerability Index score.

**Table 1.** (a) Deaths documented before hurricane Maria 13 September 2016–31 March 2017, Puerto Rico (*N* = 16,860) (b): Deaths documented during and after hurricane Maria 13 September 2017–31 March 2018, Puerto Rico (*N* = 18,510).

(a)			(b)		
Characteristic	N	%	Characteristic	N	%
Male	9181	54.45	Male	10157	54.87
Age			Age		
0–17 years	166	0.98	0–17 years	106	0.57
16–64 years	3866	22.93	16–64 years	4214	22.77
65 + years	12810	75.98	65 + years	14167	76.54
Missing	18		Missing	23	
Puerto Rican Origin	16068	95.35	Puerto Rican Origin	17615	95.23
Residence Zone			Residence Zone		
Urban	8489	50.35	Urban	9471	51.17
Rural	7969	47.27	Rural	8500	45.92
Missing	402		Missing	539	
Education			Education		
Less than high school	9237	54.81	Less than high school	9734	52.62
High School or GED	3329	19.75	High School or GED	3909	21.13
Some College	695	4.12	Some College	713	3.89
College or more	3167	18.8	College or more	3549	19.2
Missing	8		Missing	12	
Type of Death			Type of Death		
Accident	527	3.13	Accident	589	3.18
Homicide	382	2.27	Homicide	416	2.25
Natural	14736	87.52	Natural	16457	88.92
Undetermined	1087	6.45	Undetermined	905	4.89
Suicide	105	0.62	Suicide	141	0.76
Missing	23		Missing	2	

### 2.2. Data analysis

Analysis for this study can be split into three sections. First, all-cause and specific causes of mortality were assessed and risks were calculated for each census tract in the 6 month time periods before or after hurricane Maria. Second, census tracts were compared to each other in terms of having heightened risk estimates after hurricane Maria, lower risk estimates after hurricane Maria or mortality risk that stayed the same. Third, census tracts were grouped by changes in their mortality risk and were assessed by social vulnerability characteristics. All parts of this analysis are described in more detail below.

Statistical analysis software, SAS 9.4 (SAS Institute Inc.) was used for data management and statistical analysis. Crude mortality counts and mortality risks per 100 000 people were calculated for all chronic disease categories examined and all-cause mortality for the 6 months in the year before hurricane Maria (13 September 2016–31 March 2017) and for the 6 month time period following hurricane Maria (13 September 2017–31 March 2018). Crude risk difference and risk ratio were estimated comparing the post-Maria time period to the pre-Maria time period. Geocoding of decedent residence was limited to causes of death with more than 100 count difference between the two periods of comparison. These subsets of cases include the leading chronic conditions in Puerto Rico including Alzheimer’s disease, cancer, CVD, chronic Respiratory disease, diabetes, hypertension, and sepsis.

Geospatial analysis using the Getis–Ord Gi* Statistic was used to determine if mortality clusters of 6 month mortality risk per 100 000 following hurricane Maria by census tract were statistically significant and worth investigating further [18].

The Global Moran’s I, which measures spatial autocorrelation, was conducted in 500 m intervals to determine zone of indifference distance to optimize the relationship between neighboring census tracts without losing precision of the estimate. A zone of indifference of 4000 m was used for the Getis–Ord Gi* Statistic. The Getis–Ord Gi* statistic assesses the local sum of mortality risks for a census tract and the neighboring census tracts are compared to the sum of mortality risks of all census tracts. If the sum of the census tract values are much higher or much lower than the other census tracts then the difference is larger than than that that would be expected due to random chance. The *z*-score returned by Getis–Ord Gi* test statistic is a value of of 1, 2, or 3, with the highest *z*-score indicating a highly significant cluster of mortality at the 90%, 95% and 99% significance level respectively, such that the mortality risk in that census tract is much higher than surrounding tracts, and an increase that is higher than what can be attributed to random chance. Similarly, a score of −1, −2 or −3 returned from the Getis–Ord GI* statistic indicates highly significant cold spots, meaning significantly lower mortality risk than surrounding neighborhoods, with −3 being the most significant (99%) [19]. All spatial analyses were conducted in ArcGIS Pro [20].

Based on results from the Getis–Ord Gi* tests, all census tracts in Puerto Rico were classified based on their *Z* score into one of four groups: group 0 or ‘reference group’, which included census tracts that were not a significant hot/cold spot before and after hurricane Maria. Census tracts classified as group −1 were a cold spot before and after hurricane Maria. In addition, census tracts that were in a cold spot in the period before hurricane Maria and assumed a non-significant random Poisson distribution in the post-hurricane Maria period were included in group −1.(Figure 2). Census tracts classified as group 1, were defined as increased risk of chronic disease mortality, if they were not identified as a hot spot in the pre-hurricane period of analysis and identified as a hotspot at 95% significance in the 6 months post hurricane Maria. A census tract was classified as group 2, sustained high risk of chronic disease mortality, if they were a hot spot at 95% significance in both pre- and post-hurricane Maria periods. Finally, all other census tracts that did not fall into one of these categories were assumed to have a random variation of mortality in both periods, before and after hurricane Maria and were not examined further.

Multinomial logistic regression was used to model the association between census tract level social vulnerability indicators and being classified as group −1 (sustained low risk), group 1 (higher risk) or group 2 (sustained risk of mortality) with group 0 (non-significant hot/cold spots before and after) as the referent group. Social vulnerability for the three SVI themes and 13 of the indicators for social vulnerability were dichotomized into ‘high’ and ‘low’ levels at the median with ‘low’ being the reference group. Odds ratios and 95% confidence intervals were estimated to measure the association between high social vulnerability and chronic mortality risk groups with sustained or higher 6 month risk of mortality after hurricane Maria compared to group 0. The social vulnerability indicator ‘proportion of the population’ who lives in mobile homes was excluded from analysis because mobile homes are not commonly used for living in Puerto Rico and does not fit for social vulnerability in this context as it would in other parts of the United States. The whole theme on minority status was excluded from analyses because English being a second language does not relate to vulnerability in Puerto Rico the same way it does in the rest of the United States. Additionally, race is conceptualized differently in Puerto Rico and people are often misclassified in the US census data [21]. As a sensitivity analysis, final logistic regression models were used to estimate the risk of mortality hot spot classification adjusted for the proportion of the population living in multiunit structures and for proportion of the population in each census tract who were 65 years of age and older. In Puerto Rico specifically, a large proportion of condominium and apartment style living is for aging populations so these models were used to identify if there is a risk for increase mortality in multiunit living structures when adjusting for the fact that many people living in these structures may be older.

## 3. Results

Overall, there were 1650 more deaths in Puerto Rico during the 6 months following hurricane Maria than during the same period in the year before hurricane Maria. A total of 16 860 death certificates were registered in the period from 13 September 2016–31 March 2017 while 18 510 death certificates were recorded for the same time period in 2017–2018. In the post-Maria period, the majority of deaths (54.9%) were among men, similar to the pre-Maria period (table 1(b)). In the post-Maria period, most of the deceased were 65 years of age or older (76.5%), only 0.6% of deaths occurred among people under 18 years of age. (table 1(b)) Most deaths, in the post-Maria period, were attributed to natural causes (88.9%), followed by undetermined cause (4.9%), homicide, (2.3%), accidents (3.2%), and suicide (0.8%). (Table 1(b)) There were no significant differences in the relative distribution of demographic characteristics of deceased, nor in major death categories between the pre- and post-hurricane period (tables 1(a) and (b)). However, when analyzing more specific causes of death, an increase of more than 100 deaths was recorded due to CVD, Alzheimer’s, diabetes mellitus, sepsis, chronic respiratory disease, and hypertension after hurricane Maria. Minimal differences were found for asthma deaths while there were 126 fewer cancer deaths in the post-Maria period (table 2). There was a significant increase in the mortality risk in the 6 month period after hurricane Maria compared with same 6 month period a year before hurricane Maria for CVD, Alzheimer’s Disease, diabetes mellitus, sepsis, chronic respiratory disease, hypertension, end stage renal disease and suicide, as well as all-cause mortality (table 2).

**Table 2.** Crude incidence in the 6 month period before (September 2016–March 2017) and after hurricane Maria (September 2017–March 2018) by cause of death.

	Pre-Maria		Post-Maria		Difference	
Cause of Death	*N*	6 month risk per 100 000	*N*	6 month risk per 100 000	Risk difference (95% CI)	Risk ratio (95% CI)
CVD	2656	78.9	3023	92.7	13.82 (9.36, 18.28)	1.18^a^ (1.12, 1.24)
Alzheimer	1205	35.8	1437	44.1	8.28 (5.23, 11.32)	1.23^a^ (1.14, 1.33)
Diabetes mellitus	1766	52.5	1966	60.3	7.84 (4.22, 11.46)	1.15^a^ (1.08, 1.23)
Sepsis	409	12.2	566	17.4	5.21 (3.36, 7.06)	1.43^a^ (1.26, 1.62)
Chronic respiratory disease	864	25.7	985	30.2	4.55 (2.00, 7.09)	1.18^a^ (1.08, 1.29)
Hypertension	710	21.1	811	24.9	3.78 (1.47, 6.09)	1.18^a^ (1.07, 1.31)
End stage renal disease	490	14.6	556	17.1	2.50 (0.58, 4.41)	1.17^a^ (1.04, 1.32)
Suicide	104	3.1	142	4.4	1.27 (0.34, 2.20)	1.41^a^ (1.09, 1.82)
COPD	624	18.5	656	20.1	1.58 (−0.53, 3.70)	1.09 (0.98, 1.22)
Mental health Conditions	282	8.4	311	9.5	1.16 (−0.28, 2.60)	1.14 (0.97, 1.34)
Asthma	60	1.8	58	1.8	0.00 (−0.65, 0.64)	1.00 (0.70, 1.43)
Cancer	2883	85.7	2757	84.6	−1.08 (−5.52, 3.36)	0.99 (0.94, 1.04)
ALL CAUSES	16860	500.9	18510	567.8	256.03 (245.94, 266.13)	1.13^a^ (1.11, 1.15)

^a^ Significant RRs comparing post-Maria incidence risk with pre-Maria incidence risk.

The subset of decedents that were geocoded includes 10 493 deaths from the pre-Maria time period and 11 545 from the post-Maria period, a difference or excess of 1052 deaths due to the leading causes of death in Puerto Rico. As expected, compared with the overall dataset, this subset was slightly older at time of death with 82.4% being 65 and above in the pre-Maria time period and 82.61% in the post-Maria time (tables 3(a) and (b)). The subset population did not significantly differ in demographic makeup from the overall population besides in age; however it represents 63.8% of the difference in the number of deaths recorded in the pre- and post-Maria periods.

**Table 3.** (a) Deaths documented before hurricane Maria 13 September 2016–31 March 2017, Puerto Rico (*N* = 10,493) among subset of chronic disease deaths (b): deaths documented during and after hurricane Maria 13 September 2017–31 March 2018, Puerto Rico (*N* = 11,545) among subset of chronic disease deaths

(a)			(b)		
Characteristic	N	%	Characteristic	N	%

Male	5384	51.31	Male	5953	51.56
Age			Age		
0–17 years	21	0.20	0–17 years	11	0.10
18–64 years	1825	17.40	18–64 years	1996	17.29
65 + years	8645	82.40	65 + years	9537	82.61
Puerto Rican Origin	9997	95.31	Puerto Rican Origin	11030	95.58
Residence Zone			Residence Zone		
Urban	5282	50.34	Urban	5851	50.68
Rural	4960	47.27	Rural	5387	46.66
Chronic disease cause of death			Chronic Disease cause of death		
Alzheimer	1205	11.48	Alzheimer	1437	12.45
Cancer	2883	27.48	Cancer	2757	23.88
Cardiovascular disease	2656	25.31	Cardiovascular disease	3023	26.18
Chronic respiratory disease	864	8.23	Chronic respiratory disease	985	8.53
Diabetes	1766	16.83	Diabetes	1966	17.03
Hypertension	710	6.77	Hypertension	811	7.02
Sepsis	409	3.90	Sepsis	566	4.90

### 3.1. Geospatial analysis

**Figure 1.**
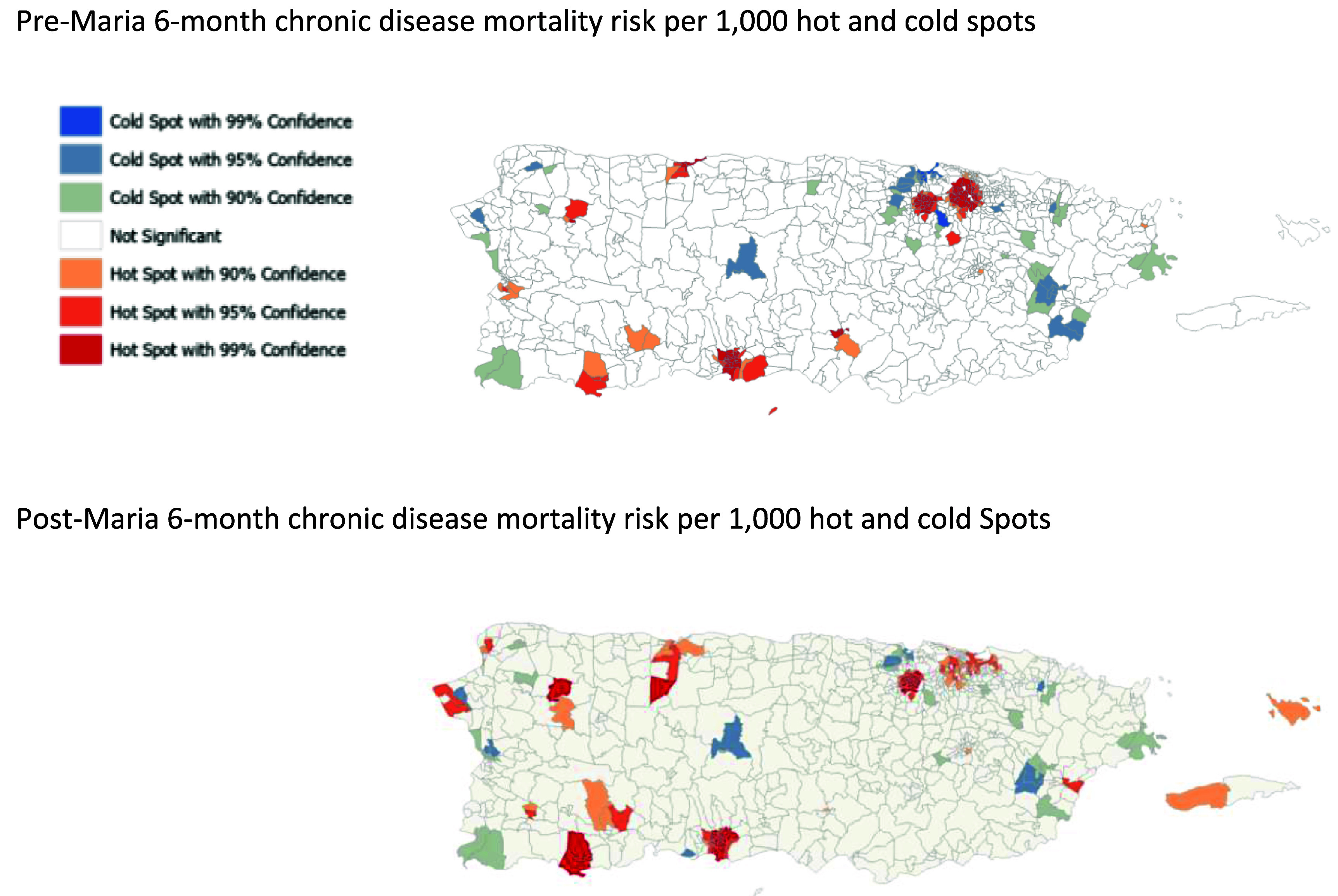
Getis–Ord Gi* statistic results for pre- and post-maria mortality risks per 100 000 people.

**Figure 2.**
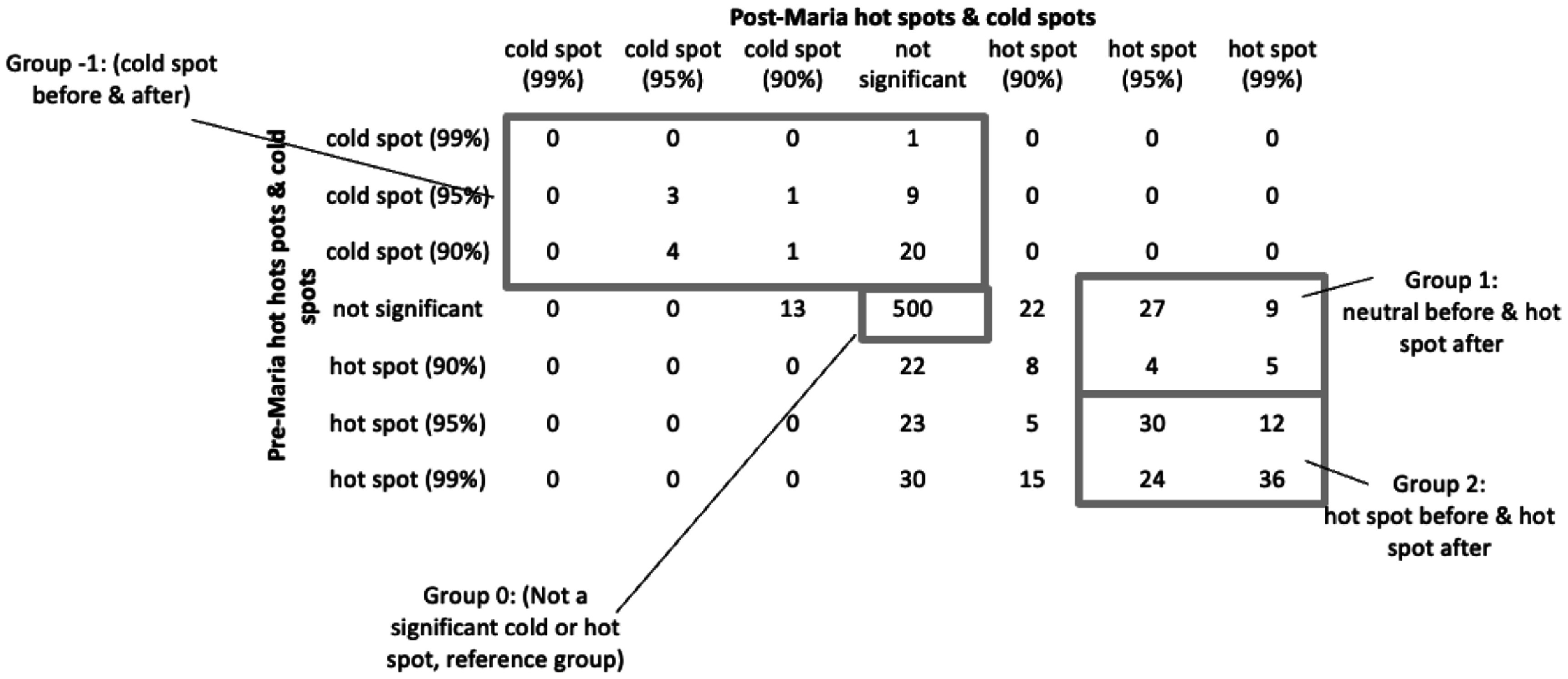
Classification of census tracts into groups for social vulnerability analysis.

The results of the geospatial analysis for selected causes of death described above in the pre-Maria and post-Maria periods are shown in figure 1. Census tracts are color coded as hot or cold spots with their corresponding *Z* statistic at a minimum of 90%, 95% and 99% significance respectively. From the crosstabulation of these results four comparison groups created are: Group 0 (reference group) with 500 census tracts were not significant hot or cold spots before or after hurricane Maria, Group −1 with 39 census tracts that were a cold spot for mortality both before and after hurricane Maria; Group 1 (increased risk) with 45 census tracts classified as not significant or hot spot with 90% significance before hurricane Maria and classified as hot spots with 95% or 99% significance in the period after hurricane Maria and; Group 2 (sustained risk) with 102 census tracts classified as hot spots with 95% or 99% significance both before and after hurricane Maria (figure 2). The community demographic profile and causes of death relative distribution did not differ significantly across groups −1, 1 or 2 however, in comparison to the reference group, the communities within census tracts in hot spots after hurricane Maria had a higher proportion of deaths due to CVD and a high proportion of deaths in the sustained higher risk were in urban census tracts (table 4). Chi-square tests for the relationship between chronic disease deaths and census tract classification indicated that there was a statistically significant association between census tract classification and chronic disease death counts.

**Table 4.** Demographic characteristics of decedents by geospatial classification.

	No census tract grouping (*n* deaths = 16,243)		Census tract group 0 (Not significant hot or cold spot (*n* deaths = 4,118))		Census tract group −1 (Sustained low risk before and after hurricane Maria, (*n* deaths = 484)		Census tract group 1 (Increased risk after hurricane Maria, (*n* deaths = 355		Census tract group 2 (Sustained high risk before and after hurricane Maria, (*n* deaths = 847)	
6 month mortality risk per 100 000										

Characteristic	*N*	%	*N*	%	*N*	%	*N*	%	*N*	%

Male	8400	51.71	2088	50.70	255	52.69	179	50.42	416	49.11
Age										
0–17 years	27	0.17	9	0.22	0	0	0	0	6	0.71
18–64 years	2800	17.24	693	16.83	95	19.63	49	13.8	184	21.72
65 + years	13416	82.60	3416	82.95	389	80.37	306	86.2	657	77.57
Puerto Rican Origin	15457	95.16	3986	96.79	475	98.14	338	95.21	773	91.26
Residence Zone										
Urban	8107	49.91	1944	47.21	341	70.45	235	66.2	715	84.42
Rural	7694	47.37	2086	50.66	133	27.48	108	30.42	119	14.05
Missing	441		85		10		12		13	
Cause of Death										
Alzheimer	1959	12.06	497	12.07	57	11.78	45	12.68	85	10.04
Cancer	4116	25.34	1079	26.20	143	29.55	91	25.63	212	25.03
Cardiovascular Disease	4168	25.66	1058	26.59	112	23.14	103	29.01	238	28.1
Chronic Respiratory Disease	1373	8.45	317	7.70	43	8.88	35	9.86	81	9.56
Diabetes	2765	17.02	715	17.36	74	15.29	45	12.68	133	15.7
Hypertension	1164	7.17	262	6.36	26	5.37	19	5.35	50	5.9
Sepsis	697	4.29	187	4.54	29	5.99	17	4.79	45	5.31

Comparing geospatial classification groups by social vulnerability index components for Themes 1, 2 and 4, the housing composition and disability status were the only characteristics significantly related with geospatial classification for group 1 with increased risk of mortality after the hurricane. Housing and transportation were significantly associated with spatial classification as a sustained high risk of mortality area. Theme 3 was excluded from analysis as it refers mainly to minority status, not a useful indicator in Puerto Rico since the entire population is considered a ‘minority’ in the U.S. Census statistics The only vulnerability indicators associated with a higher risk or sustained risk of mortality were census tracts with a high proportion of the population 65 years or older, proportion of the housing structures with 10 or more household units and proportion of households with no vehicle (table 5) The risk of mortality adjusting for these two vulnerability indicators did not change significantly from the unadjusted models (supplemental table 2).

**Table 5.** Crude odds ratio of census tract hot spot classification comparing high social vulnerability to low vulnerability.

	Group −1 (Sustained low risk)	Group 1 (Increased risk after hurricane)	Group 2 (Sustained high risk)
**Theme 1: Socioeconomic**	0.99 (0.64, 1.52)	0.93 (0.51, 1.72)	0.99 (0.64, 1.52)

Poverty	0.62 (0.32, 1.21)	0.87 (0.47, 1.60)	1.29 (0.84, 2.00)
Unemployment	1.00 (0.52, 1.92)	1.58 (0.85, 2.93)	1.07 (0.70, 1.65)
Income	0.69 (0.36, 1.35)	1.27 (0.69, 2.34)	1.41 (0.92, 2.16)
No High School Diploma	1.14 (0.59, 2.19)	1.12 (0.61, 2.06)	0.92 (0.60, 1.42)

**Theme 2: House Comp & Disability**	0.64 (0.17, 0.70)	2.67 (1.32, 5.39)^a^	1.17 (0.76, 1.81)^a^

Aged 65 or older	0.27 (0.12, 0.62)	2.45 (1.29, 4.67)^a^	2.05 (1.32, 3.20)^a^
Aged 17 or younger	0.87 (0.45, 1.67)	1.50 (0.79, 2.82)	0.49 (0.32, 0.76)
Civilian with a disability	0.73 (0.38, 1.40)	0.90 (0.49, 1.65)	0.74 (0.48, 1.14)
Single-parent household	1.53 (0.79, 2.97)	1.76 (0.94, 3.29)	0.95 (0.62, 1.45)

**Theme 4: Housing and Transportation**	1.10 (0.57, 2.12)	1.29 (0.70, 2.40)	1.58 (1.02, 2.47)^a^

Multi-unit structures	0.40 (0.19, 0.87)	3.71 (1.87, 7.34)^a^	4.63 (2.82, 7.61)^a^
Crowding	3.33 (1.50, 7.38)	0.16 (0.07, 0.36)	0.93 (0.61, 1.42)
No vehicles	1.10 (0.57, 2.12)	2.57 (1.35, 4.91)^a^	2.16 (1.38, 3.36)^a^
Group quarters	1.02 (0.53, 1.96)	0.90 (0.49, 1.66)	0.60 (0.39, 0.92)

^a^ Significant with confidence interval not including the null of 1.

**Figure 3.**
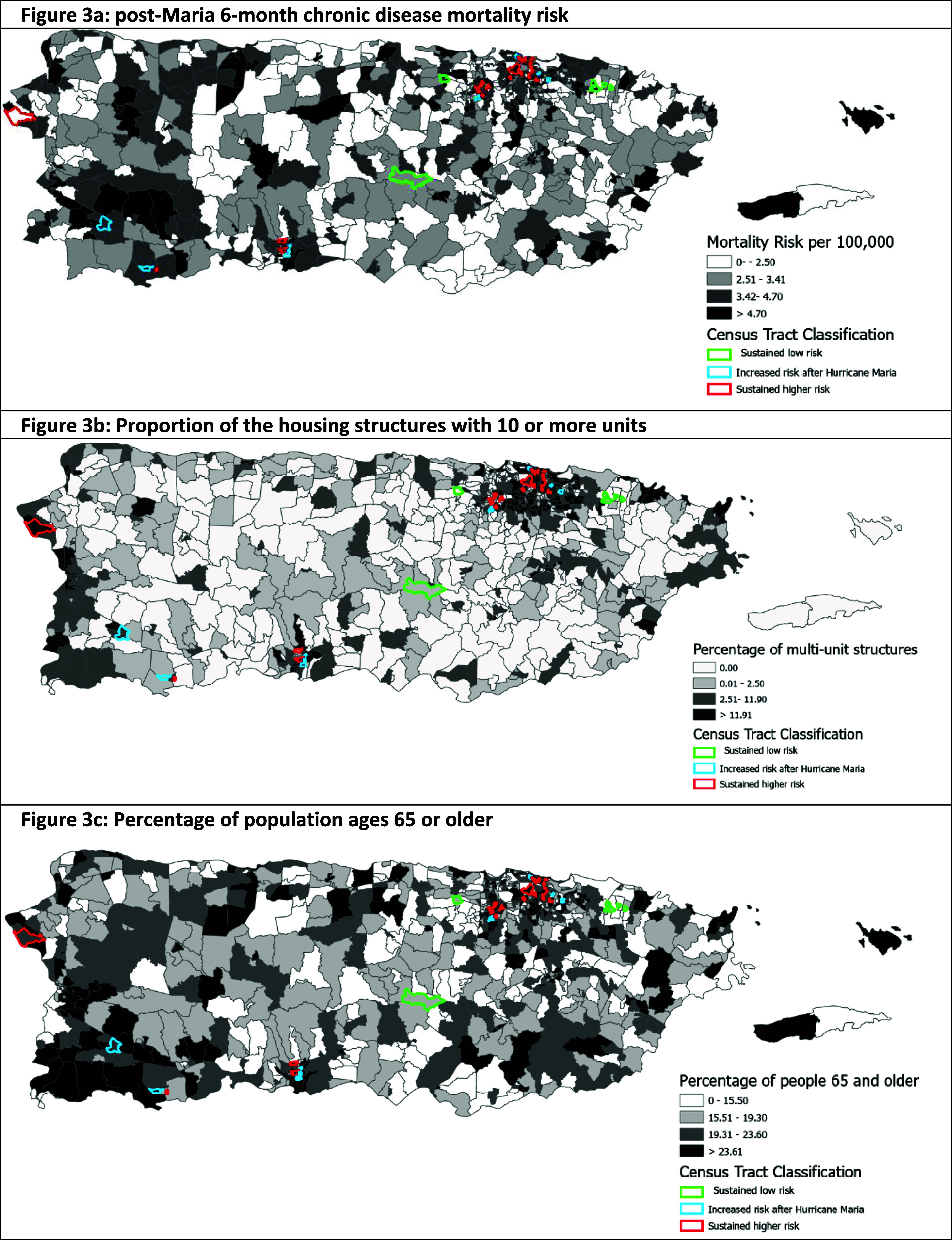
(a). Post-Maria 6 month chronic disease mortality risk.(b). Proportion of the housing structures with 10 or more units.(c). Percentage of population ages 65 or older.

Figure 3(a) shows the mortality risk for chronic diseases after hurricane Maria and the classification of mortality risk based on the geospatial analysis. Figures 3(b) and (c) show the classification of census tracts according to the proportion of housing structures with 10 or more units and proportion of the population 65 years of age or older respectively. Group 1 had a significant increased mortality risk after the hurricane compared to the reference group in the housing composition vulnerability characteristic (OR: 2.67, 95% CI 1.32, 5.39). Housing and transportation were significantly associated with increased risk of mortality for those census tracts with increased risk before and after the hurricane. (OR: 4.63, 95% CI 2.82, 7.61)

## 4. Discussion

The initial findings suggest an excess number of deaths occurred in the post-Maria period compared to a similar period a year before the hurricane. While our estimate is more conservative, these findings are similar to the study commissioned by the government of Puerto Rico which estimated excess mortality post-hurricane Maria [3]. In the 6 months following hurricane Maria there were increases in mortality risk for CVD, Alzheimer’s, diabetes, sepsis, chronic respiratory disease, hypertension, and all-cause mortality. These findings are consistent with other studies on the impacts of hurricanes on mortality which have found that exacerbations of pre-existing chronic conditions are common causes of mortality after a hurricane [22, 23]. Our study also identified 50, 85 and 62 census tracts that were hot spots for chronic disease mortality risk with 90%, 95% and 99% significance respectively in the 6 months following hurricane Maria. Comparing hot spots before and after hurricane Maria, 45 census tracts had an increased risk of being a hotspot for mortality risk after hurricane Maria and 102 census tracts had a sustained high risk before and after.

Examining community level characteristics associated with vulnerability to disasters, having a high proportion of the population that is 65 years and older, having a high proportion of houses being multiunit structures and having a high proportion of households with no vehicle was associated with census tracts experiencing sustained high risk and increased risk of chronic disease mortality after hurricane Maria. Interestingly, some vulnerability characteristics did not follow the trends expected of them. For example, overcrowding was associated with lower levels of mortality before and after hurricane Maria (group −1) and was associated with a decreased risk of mortality after the storm compared to the year before hurricane Maria (group 1). It was expected that overcrowding would be associated with either increased risk of mortality after the storm (group 1) or sustained higher mortality both before and after hurricane Maria (group 2). This vulnerability indicator measures as the number of households with more people than rooms may be context-dependent and more applicable to community recovery after a disaster. However in our study, overcrowding could be indicative of a greater a greater support network for individuals with chronic disease conditions compared to households with one or two people living in it.

An immediate impact of Hurricane Maria was the large migration from Puerto Rico to the U.S. mainland, mostly to the state of Florida where there is already a large population of Puerto Rican residents. According to the US Census Bureau estimates, there was a 4.4% decrease in the total population of Puerto Rico between 2017 and 2018 [24]Our analysis sought to account for this shift by using denominator data on the population from both 2017 and 2018 after hurricane Maria. An analysis of mobile phones and social media data suggests a larger proportion of internal population displacement occurred, between 8% to 17% from rural to urban areas of the Island [25]. The internal population displacement and out-migration to the US mainland may explain in part the significant decrease observed in the incidence of cancer post Hurricane Maria attributed to a delay in cancer diagnosis and treatment in Puerto Rico [26],It is possible that differential out-migration occurred, with people with cancer being more likely to leave to get care for their condition than other chronic diseases. On the other hand, a retrospective cohort study of Puerto Rican women with gynecological cancers and treatment interruption after Hurricane Maria found a significant excess risk of death after a follow-up to December 2019 which extends beyond the time period in the present study and may explain longer term increases in cancer mortality observed than our study was able to capture [27].

There are limitations to our study. Our study was limited to being able to examine death records only from the 6 months after hurricane Maria and the same 6 months in the year before due to funding constraints. For this reason, we rely on chronic disease mortality risks from the year before as the comparison which may not be consistent with general trends for mortality in Puerto Rico over the many years before Maria, however there were no other major hurricanes or events in the year prior to hurricane Maria so the year before serves as a good proxy for trends in mortality in Puerto Rico outside of a disaster context. Additionally, the spatial analysis of social vulnerability examines the impacts of vulnerability on specific groupings of mortality clusters only in the years before and after Maria with varying alpha levels from the Getis–Ord Gi* statistic. While this is informative for this specific hurricane, this may not be generalizable to other hurricanes that have occurred or will occur in Puerto Rico. Because the data is de-identified, our study does not involve any methods to collect data on movement within Puerto Rico from the year before Maria to the year after or more qualitative narrative information. Additionally, death certificate data is subject to the biases of whoever fills out the death certificate. It is possible that the increased workload and lack of medical staff led to death certificates being filled in with less detail in the months immediately following hurricane Maria. Another limitation of this study is that it does not capture deaths of people who left Puerto Rico after h Maria. It is possible that people with serious chronic diseases left Puerto Rico due to hurricane Maria, and those that died outside of the territory would not be captured in this dataset. Additionally, due to funding constraints, our spatial analysis was limited for specific chronic disease deaths that are known to be common causes of death in Puerto Rico.

Despite the limitations, the findings from this study are significant to public health due to the identification of certain chronic conditions that experienced increases in mortality risk following hurricane Maria in Puerto Rico. Additionally, this study identified geographic areas that may be at higher risk of increases in mortality following hurricanes through spatially examining the distribution of mortality and mapping out vulnerability characteristics that were associated with increased risks in mortality. This study demonstrates that some characteristics as defined by CDC’s Social Vulnerability Index, including proportion of households that are multiunit structures and proportion of the population that is 65 and older may be useful for identifying communities at higher risk of chronic disease mortality after a hurricane. All risk factors examined in this study were at the community level so further research is needed to identify individual-level risk and protective factors associated with chronic disease mortality following a disaster in Puerto Rico.

## 5. Conclusions

Our key findings describe the post-hurricane death toll in Puerto Rico. In the 6 months examined during 2017–2018, there were 18 510 deaths recorded in Puerto Rico compared with 16 860 deaths in the pre-Maria period of 2016–2017. Our study showed that there is limited documentation in the post-disaster death records to determine if any death may have occurred directly or indirectly due to a disaster. Following hurricane Maria there were increases in 6 month Mortality risk for CVD, Alzheimer’s, diabetes, sepsis, chronic respiratory disease, hypertension, and all-cause mortality. Of important note is the decrease in the number of deaths due to cancer after hurricane Maria. This finding deserves further study to determine possible factors related to population displacement after a disaster. Hot and cold spots of mortality risk following hurricane Maria were mapped to demonstrate where the highest increases in mortality occurred. The findings from our hotspot analysis indicate that communities with a high proportion of the population who is 65 and older and areas with a high proportion of households that are living in multiunit structures may be at higher risk of experiencing higher chronic disease mortality following a disaster of the magnitude of hurricane Maria. The results from this study can also inform efforts to better communicate proper post-disaster death certificate methods to medical professionals. In future disasters where health services are severely affected, people with chronic diseases should be prioritized in the response and recovery phase of disaster management.

## Data Availability

The data cannot be made publicly available upon publication because they contain sensitive personal information. The data that support the findings of this study are available upon reasonable request from the authors.
